# Structured Literature Review of the Impact of Using Cognitive-behavioural Therapy and Interpersonal Therapy On Depressive Symptoms in People Living With HIV/AIDS.

**DOI:** 10.1192/bjo.2025.10161

**Published:** 2025-06-20

**Authors:** June Kilonzo

**Affiliations:** Cardiff University, Wales, United Kingdom

## Abstract

**Aims:** A diagnosis of depression is three times more likely to occur in people living with HIV/AIDS. Management is imperative,as it not only ensures reduced depressive symptoms but also improves quality of life. Interpersonal psychotherapy (IPT) and cognitive-behavioural therapy (CBT) have been linked to reduced depressive symptoms. This paper will investigate to what degree both treatment options can help reduce depressive symptoms in people living with HIV/AIDS.

**Methods:** Randomized controlled trial articles from the Cardiff University repository, PsycARTICLES, PsycINFO, Cinahl, and PubMed databases were collected and dated not later than ten years from 2024. The participants were individuals with an HIV/AIDS diagnosis and depression as a comorbidity. They also had been exposed to either IPT or CBT psychotherapy. The articles were screened, and data from19 articles with 4805 participants were extracted.

**Results:** Findings showed that CBT reduced depressive symptoms, and its effects were visible in the long term, which involves more than six months. Similarly, IPT was found to have long-term effects, but the difference in the symptoms in IPT was minimal compared with CBT. No significant difference in the efficiency of either therapy was observed.

**Conclusion:** Both the psychotherapy modalities were found to have positive impact by reducing depressive symptoms. There is a need for more research into improving the psychotherapy intervention time and mode of delivery.

